# Comorbidity and mortality in adults with juvenile idiopathic arthritis: a nationwide Norwegian register-based matched cohort study

**DOI:** 10.1093/rheumatology/keag507

**Published:** 2026-09-15

**Authors:** Imane Bardan, Lene M Sundbakk, Joe Sexton, Tore K Kvien, Øyvind Molberg, Eirik K Kristianslund, Sella A Provan, Anna-Birgitte Aga

**Affiliations:** Department of Rheumatology, Oslo University Hospital, Oslo, Norway; Center for Treatment of Rheumatic and Musculoskeletal Diseases (REMEDY), Diakonhjemmet Hospital, Oslo, Norway; Institute of Clinical Medicine, University of Oslo, Oslo, Norway; Center for Treatment of Rheumatic and Musculoskeletal Diseases (REMEDY), Diakonhjemmet Hospital, Oslo, Norway; Center for Treatment of Rheumatic and Musculoskeletal Diseases (REMEDY), Diakonhjemmet Hospital, Oslo, Norway; Center for Treatment of Rheumatic and Musculoskeletal Diseases (REMEDY), Diakonhjemmet Hospital, Oslo, Norway; Institute of Clinical Medicine, University of Oslo, Oslo, Norway; Department of Rheumatology, Oslo University Hospital, Oslo, Norway; Institute of Clinical Medicine, University of Oslo, Oslo, Norway; Center for Treatment of Rheumatic and Musculoskeletal Diseases (REMEDY), Diakonhjemmet Hospital, Oslo, Norway; Center for Treatment of Rheumatic and Musculoskeletal Diseases (REMEDY), Diakonhjemmet Hospital, Oslo, Norway; Section for Public Health and Sports Sciences, University of Inland Norway, Elverum, Norway; Department of Rheumatology, Oslo University Hospital, Oslo, Norway; Center for Treatment of Rheumatic and Musculoskeletal Diseases (REMEDY), Diakonhjemmet Hospital, Oslo, Norway

**Keywords:** juvenile idiopathic arthritis, comorbidities, mortality

## Abstract

**Objectives:**

This population-based study assesses comorbidities, autoimmune conditions and all-cause mortality in [1] adults with juvenile idiopathic arthritis (JIA) against general population comparators and [2] adults with JIA with *vs* without recent disease-modifying anti-rheumatic drug (DMARD) exposure.

**Methods:**

We included adults who, between 2009 and 2024, had ≥2 specialist health contacts with a JIA-specific ICD-10 code in the mandatory Norwegian Patient Registry (NPR). Each case was matched randomly, by age, gender and county of residence, to 10 general population comparators at the time of receiving the second JIA diagnosis in adulthood. We compared 5-year retrospective comorbidity prevalences in descriptive analyses and prospective all-cause mortality in [1] adults with JIA *vs* comparators and [2] adult JIA with *vs* without recent DMARD exposure.

**Results:**

Adults with JIA (*N* = 2932) had higher prevalence than comparators (*N* = 29 097) of ischemic heart disease excluding myocardial infarction [JIA *vs* comparators (%): 1.0 *vs* 0.6], hypertension (3.6 *vs* 1.4), chronic kidney disease (0.5 *vs* 0.2), type 1 diabetes (1.7 *vs* 0.7), coeliac disease (1.4 *vs* 0.7), autoimmune thyroiditis (0.3 *vs* 0.1) and autoimmune alopecia (0.4 *vs* 0.1). Prospective all-cause mortality was higher in JIA than comparators (mortality rate 2.4 *vs* 1.8 per 1000 person-years). In adult JIA, prevalences of comorbidities and autoimmune conditions as well as all-cause mortality was similar in those with and without recent DMARD exposure.

**Conclusion:**

In this study we found an increased 5-year retrospective prevalence of several comorbidities and increased all-cause mortality in adults with JIA compared with population controls.

*Rheumatology* key messages

- Adults with juvenile idiopathic arthritis (JIA) had a higher prevalence of comorbidities and autoimmune conditions than the general population.
- All-cause mortality was higher in adults with JIA compared to those without JIA.
- Comorbidity prevalences and all-cause mortality were equal in adults with JIA with versus without recent/current DMARD exposure.

## Introduction

Adults with juvenile idiopathic arthritis (JIA) carry the burden of cumulative disease activity. JIA shares chronic autoimmune joint inflammation with adult-onset inflammatory joint diseases (IJDs) [1]. These diseases are associated with an increased risk of major comorbidities including cardiovascular disease (CVD) [2, 3], chronic kidney disease (CKD) [4] and type 2 diabetes mellitus (DM) [5], which often co-occur due to overlapping predisposing factors. To our knowledge, no previous study has explored the risk of these comorbidities in adults with JIA compared with matched comparators.

Chronic inflammation increases the risk of CVD and CKD, largely due to accelerated atherosclerosis [6]. While studies exploring CVD in adults with JIA are scarce, they indicate excess risk of hypertension and premature atherosclerosis [7–9]. Hypertension, atherosclerosis and use of non-steroidal anti-inflammatory drugs (NSAIDs) are factors that also increase risk for CKD [10]. It is, however, uncertain whether adults with JIA are at increased risk of CKD. Cumulative evidence suggests that the excess risk for type 2 DM in patients with rheumatoid arthritis may partly be driven by chronic inflammation [5]. No previous study has explored the risk of type 2 DM in adults with non-systemic JIA. Reasonably, reduced inflammation may reduce the risk of these comorbidities, and there is some evidence suggesting that disease-modifying anti-rheumatic drugs (DMARDs) may reduce the risk of CVD [11, 12], type 2 DM [13] and CKD [14, 15].

JIA is defined as chronic, immune-mediated arthritis with onset before the age of 16 years and is classified into seven categories by the International League of Associations for Rheumatology (ILAR) [1, 16]. Several studies show that JIA is not a disease confined to childhood, as around 50% of individuals with JIA have active disease in adulthood [17–20]. Some JIA phenotypes are closely linked to defined autoimmune conditions, including uveitis, psoriasis and inflammatory bowel disease (IBD), which are considered as extraarticular manifestations of JIA rather than JIA-associated disorders [16]. However, studies in juvenile settings indicate that JIA comes with increased risk for several autoimmune diseases [21]. There is an increased risk of type 1 DM, likely due to the shared genetic risk factors [22]. To date, there are no studies on this issue in adults with JIA.

Studies suggest a higher mortality compared with the general population; however, these were published two decades ago [23, 24] and current evidence is lacking. Furthermore, recent studies have suggested a reduction in mortality in adults with IJDs treated with DMARDs [25, 26]. Based on the increased mortality [27–29] and associations between inflammation and cardiovascular comorbidities, CKD and type 2 DM in adults with IJDs [2–5], we aimed for the first time to explore the prevalence of common non-autoimmune and autoimmune comorbidities as well as the all-cause mortality in adults with JIA compared with general population comparators. Additionally, we explored whether these outcomes are different in adult JIA patients with *vs* without recent or current DMARD exposure.

## Methods

### Data materials

We used data from the nationwide Norwegian Patient Registry (NPR) to identify adults with JIA between 1 January 2009 and 1 January 2025. NPR includes data from 2008 and onwards on patient contacts in public special healthcare services, including contract specialists [30]. Data from 2008–2009 is incomplete and was discarded. Diagnoses are coded using the International Statistical Classification of Diseases and Related Health Problems, 10th Revision (ICD-10) [31], and procedures are registered with procedure codes.

Data was linked to the Norwegian Prescribed Drug Registry, which contains information on all dispensed prescription medication in Norwegian pharmacies since 2004. Information on drugs administered at hospitals was acquired using procedure codes registered in NPR linked to Anatomical Therapeutic Chemical (ATC) codes for the administered drugs. For deceased individuals, time of death was acquired by register linkage to the Cause of Death Registry. Data on comparators as well as data on education and county of residence was acquired from Statistics Norway.

The study complies with the Declaration of Helsinki. Data materials are based on anonymized registry data. Ethical approval has been given by the Regional Committees for Medical and Health Research Ethics South-East Norway (reference number: 68 124). Data storage was approved by the Data Inspectorate.

### Adult JIA population

The adult JIA population consisted of individuals ≥18 years with at least two ICD-10 diagnoses of JIA, corresponding to codes *M08* or *M09*, registered in the NPR after reaching adulthood. The two codes had to represent the patient’s first registered diagnoses within the category of IJDs. Patients with Still’s disease, identified as those whose first two IJD diagnoses in the NPR were coded as *M08.2*, were excluded. Adults (≥18 years) entered the study at the date of their second registered JIA diagnosis in the NPR, defined as the index date. As the NPR was established in 2009, individuals who turned 18 before 2009 were included only if they received two JIA diagnoses in the NPR after 2009, implying continued follow-up in specialist healthcare services.

### General population comparators

At the index date, patients were matched 1:10 to comparators without JIA by age, gender and centrality index. The centrality index is a measure developed by Statistics Norway and is used to classify municipalities in Norway into six categories based on urbanity, and access and proximity to services. Each municipality is given a coded value from 1 to 6, where lower values indicate more central municipalities. For instance, the capital of Norway, Oslo, has a value of 1. Matching was done by Statistics Norway.

### Study design and length of follow-up

#### Retrospective analyses

Patients and comparators had a comorbidity if a given ICD-10 code was registered during a five-year period retrospectively to the index date. To ensure a look-back of at least five years for all patients and comparators, and since we used NPR data from 2009 and onwards, patients and comparators were required to have an index date from 1 January 2014. Consequently, for analyses on Charlson Comorbidity Index (CCI) and prevalences of comorbidities and autoimmune conditions, the index date was redefined to 1 January 2014 for individuals with an index date prior to 2014, adding equal time to patients and their associated comparators. This approach was chosen to maximize the number of eligible patients, particularly those with longstanding disease. Patients and comparators who died between 2009 and 2014 were excluded from the analyses.

For analyses on drug use, use of DMARDs and NSAIDs was defined as drugs dispensed at pharmacies or administered at hospitals during the year preceding the index date.

#### Prospective analyses

For mortality analyses, time under risk was defined as time from the index date to (i) death or (ii) the end of data extraction (1 Janusry 2025), whichever occurred first.

### Outcome measures

#### Retrospective analyses

##### Prevalence of comorbidities

We calculated 5-year retrospective prevalence of selected common comorbidities: myocardial infarction (MI), ischemic heart disease other than MI, hypertension, congestive heart failure (CHF), non-hemorrhagic stroke, CKD and type 2 DM. ICD-10 codes used to define the comorbidities are listed in Supplementary Table S1.

##### Prevalence of autoimmune conditions

The 5-year prevalence of the following comorbidities was calculated: type 1 DM, coeliac disease, autoimmune thyroiditis, autoimmune alopecia, IBD, multiple sclerosis, psoriasis and uveitis. ICD-10 codes used to define the autoimmune conditions are listed in Supplementary Table S1.

##### Charlson Comorbidity Index (CCI)

The Charlson Comorbidity Index (CCI) describes the comorbidity burden in individuals based on the presence and severity of selected comorbidities [32]. The score predicts 10-year mortality risk [32]. A modified CCI score, based on 5-year retrospective data, was calculated for adults with JIA and their matched comparators. The comorbidities included in CCI are the following: previous MI, CHF, peripheral vascular disease, cerebrovascular accident or transient ischemic attack (TIA), dementia, chronic pulmonary disease, rheumatic disease, peptic ulcer disease, liver disease, DM, hemiplegia, moderate-to-severe CKD, solid tumour, leukaemia, lymphoma and AIDS [33]. We modified the score by excluding rheumatic disease, as JIA patients are more likely to be diagnosed with other rheumatic diseases than comparators. CCI was calculated for each individual and summary statistics are presented for 10-year age groups, starting from 25 years of age. The score included ICD-10 codes registered during an observation time of five years retrospectively to the index date.

##### Use of disease-modifying anti-rheumatic drugs (DMARDs)

The number of patients exposed to DMARDs during the year prior to the index date was calculated. Use of DMARDs was defined as (i) dispensing of DMARD prescriptions in a Norwegian pharmacy registered in The Norwegian Prescribed Drug Registry and/or (ii) DMARDs administered at hospitals registered as ATC codes for DMARDs in NPR during the year preceding the index date. DMARDs were further divided into (i) conventional synthetic DMARDs (csDMARDs), (ii) biological DMARDs (bDMARDs) and (iii) targeted synthetic DMARDs (tsDMARDs).

#### Prospective analyses

##### All-cause mortality

The mortality rate was calculated 5 and 10 years after the index date.

### Statistical analysis

Descriptive statistics included: (i) demographics at index date; (ii) 5-year retrospective prevalence of comorbidities and autoimmune conditions in JIA and matched comparators; and (iii) 1-year retrospective DMARD and NSAID use in JIA and comparators. Categorical variables were presented as frequencies with percentages. Continuous variables were presented as mean value with standard deviation (SD). CCI is an ordinal variable ranging from 0 to 37; however, mean (SD) CCI-scores were calculated in addition to the medians with interquartile range (IQR) to better illustrate differences between JIA and comparators, as the results are skewed towards 0. Prospective analyses included all-cause mortality, which was reported as mortality rates per 1000 person-years at risk.

To assess differences between JIA and comparators, we used the following statistical methods. Differences in demographics were analysed using chi-squared test for categorical variables and the independent samples *t* test for continuous variables. The Mann–Whitney *U* test was used to assess differences in CCI score. Differences in 5-year retrospective comorbidity prevalences were analysed using the chi-squared test. Bonferroni correction was conducted to adjust for multiple testing. Prospective all-cause mortality was compared between JIA and comparators using the log-rank test and a Cox proportional hazards regression model to estimate the hazard ratio (HR) and the 95% confidence interval. A Cox regression model adjusted for educational level and centrality index was conducted to explore the potential confounding effect of these variables.

We used logistic regression adjusted for age and gender to evaluate the 5-year retrospective prevalences of comorbidities and autoimmune conditions in JIA patients with *vs* without recent or current DMARD treatment. To compare CCI in adult JIA with *vs* without recent or current DMARD exposure, we performed age- and gender-stratified analyses, using the Mann–Whitney *U* test. Cox proportional hazards regression models including (i) age, gender and DMARD exposure (as defined previously), (ii) age, gender, educational level, centrality index and DMARD exposure and (iii) age, gender, retrospective CCI-score and DMARD exposure were used to assess the risk of all-cause mortality in the two groups.

We performed complete case analyses, as there was only 2% missing data, which was considered missing at random. The significance level (P) was <0.05 for all statistical analyses, which were conducted in StataBE 18 (64-bit) for Windows.

## Results

### Patient selection and demographics

We included 2932 adult patients with JIA and 29 097 comparators in the analyses of drug use and mortality. For the analyses of CCI and comorbidities, 2896 adult JIA patients and 28 966 comparators were included. Patient selection is illustrated in Fig. 1. As intended by the matching, age, gender distribution or centrality index did not differ between patients and comparators, as shown in Table 1. Excluding the first two years, when the majority of prevalent adult JIA cases were included in the study cohort, we had median 149 new cases who fulfilled the defined criteria for ‘adult with JIA’ every year throughout the study period.

**Figure 1:**
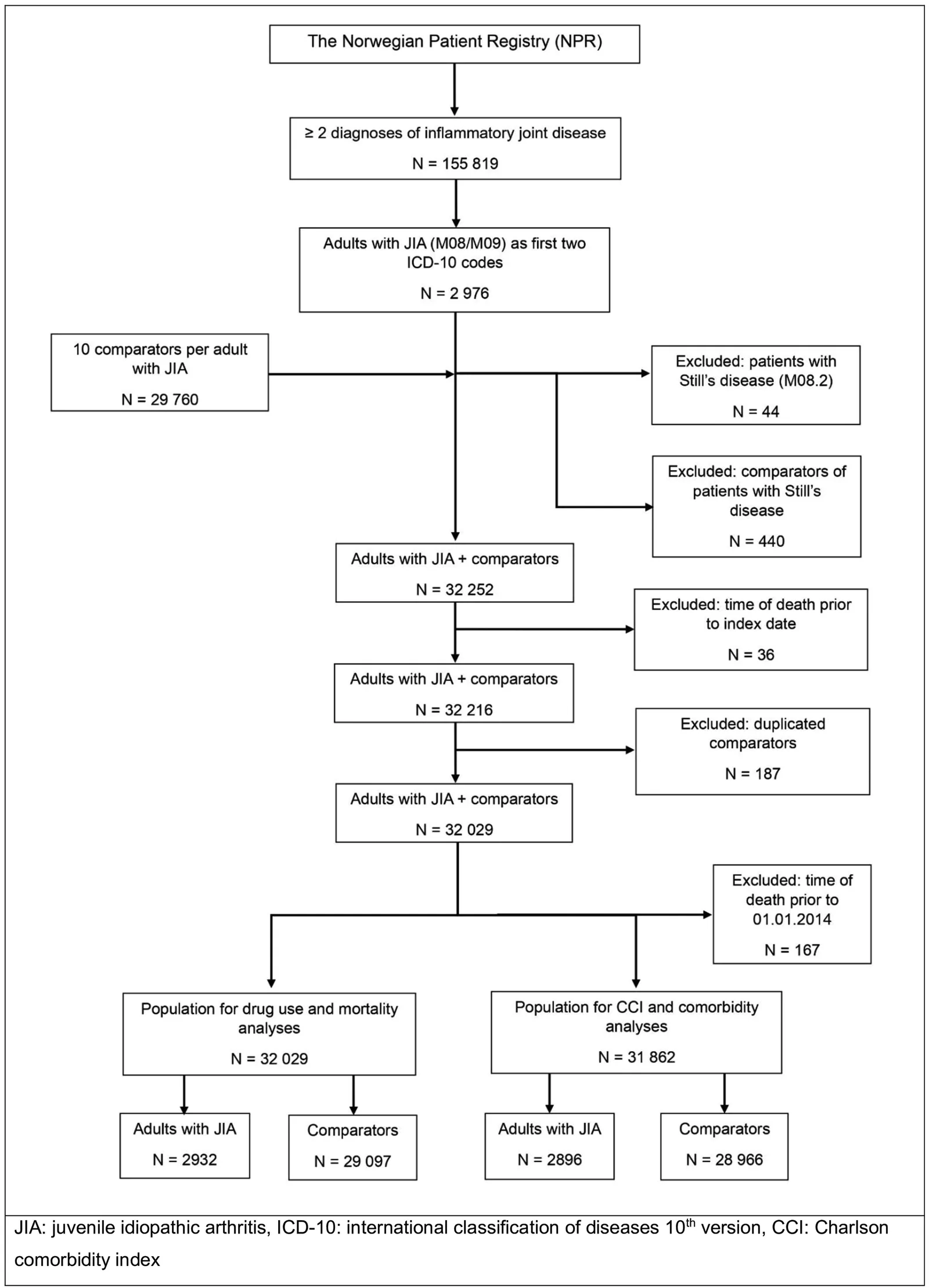
Flowchart of study population

**Table 1:** Demographics at index date and use of DMARDs at index date.

	Adults with JIA N = 2932	**Comparators N = 29** **097**
**Age, years (mean (SD))**	27.30 (13.99)	27.29 (13.92)
**Female gender, *n* (%)**	2076 (70.8)	20 597 (70.8)
**Educational level, *n* (%)**		
Secondary school graduate	1627 (55.5)	16 101 (55.3)
College graduate	772 (26.3)	7177 (24.7)
University/higher education graduate	493 (16.8)	5107 (17.6)
Missing	40 (1.4)	278 (1.0)
**Centrality index, median (IQR)**	**3 (2–4)** *n* = 2656	**3 (2–4)** *n* = 26 231
**DMARD prescription,** ^a^ ***n* (%)**	**1532/2932 (52.3)**	**48/29** **096 (0.2)**
csDMARDs monotherapy	627 (21.4)	29 (0.1)
bDMARDs +/− csDMARD	900 (30.7)	19 (0.1)
tsDMARDs^b^	5 (0.0)	0 (0.0)
**NSAID prescription,^a^ *n* (%)**	**1493/2932 (50.9)**	**4022/29** **096 (13.8)**

a Drug prescriptions dispensed or drugs administered at hospitals during the year preceding the index date.

b Two patients were exposed to both tsDMARD and bDMARD during the year prior to index date; these are only counted in the tsDMARD group.

JIA: juvenile idiopathic arthritis; DMARD: disease-modifying anti-rheumatic drug; csDMARD: conventional synthetic DMARD; bDMARD: biologic DMARD; JAKi: janus kinase inhibitor; NSAID: non-steroidal anti-inflammatory drug. Significant values in bold. Significance level is *P* < 0.05.

### Use of DMARDs

At the index date, 52.3% of the adult JIA patients had used DMARDs during the past year. The percentage exposed to csDMARDs without bDMARDs was 21.4 and to bDMARDs ± csDMARDs was 30.7. Five adults with JIA had used JAKi, of whom one had been exposed to both JAKi and bDMARD during the year prior to index date. An overview of drugs within the DMARD subgroups is given in Supplementary Table S2. Use of prescribed NSAIDs was registered in 50.9% of the JIA population and 13.8% of comparators at index date (Table 1).

### Comorbidities

Adults with JIA had significantly higher 5-year retrospective prevalence (%) of ischemic heart disease excluding acute MI (1.0 *vs* 0.6), hypertension (3.6 *vs* 1.4) and CKD (0.5 *vs* 0.2). The 5-year retrospective prevalence of MI, CHF, non-haemorrhagic stroke and DM 2 were similar in JIA and comparators (Table 2, Fig. 2).

**Figure 2:**
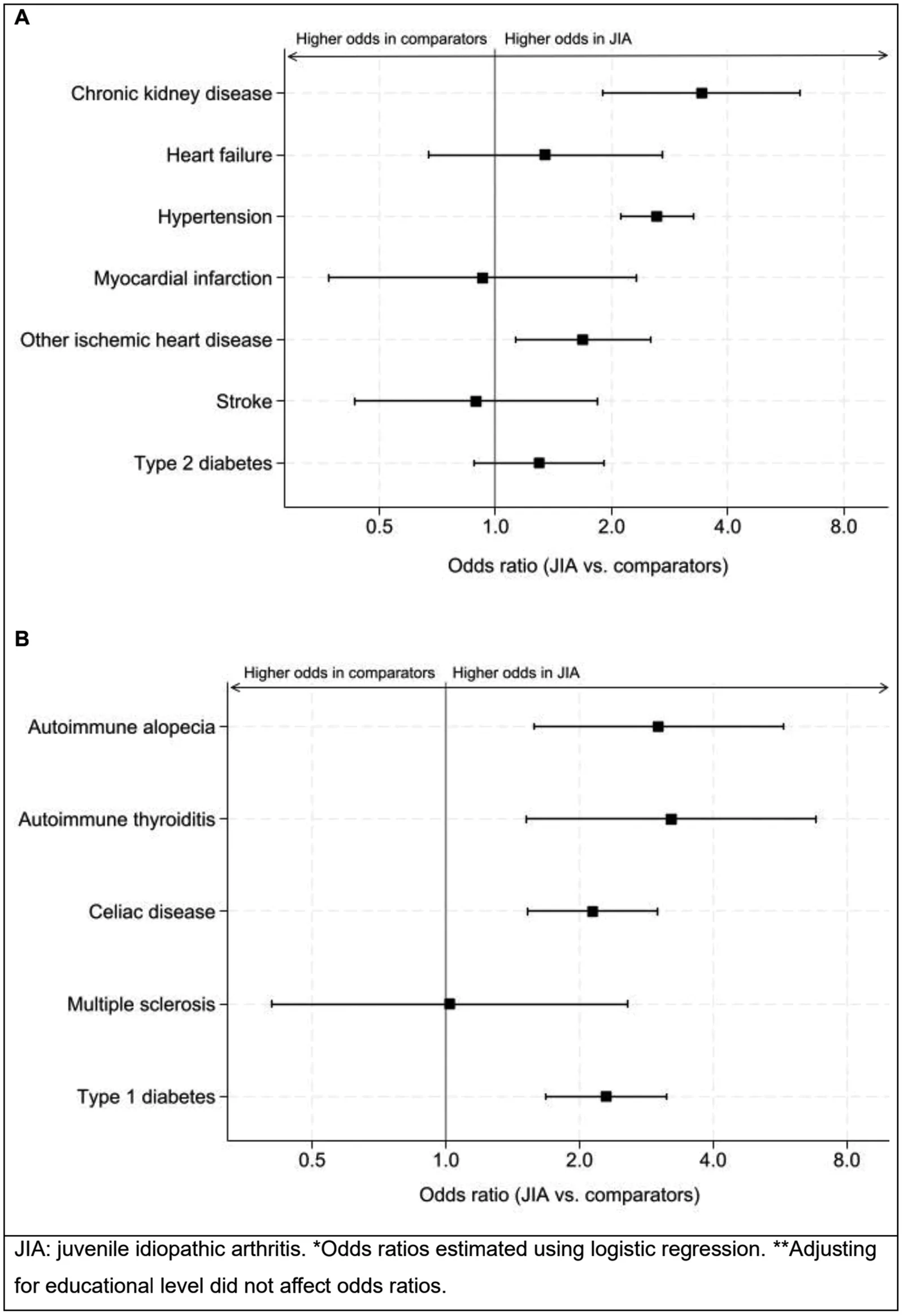
Five-year odds ratios for common comorbidities (**A**) and autoimmune diseases (**B**) in adults with JIA *vs* comparators based on retrospective data

**Table 2:** Prevalence [*n* (%)] of comorbidities in adults with JIA and comparators 5 years retrospectively.

	Adults with JIA N = 2896	**Comparators N = 28** **966**	***P* value** ^a^
**Comorbidities**			
Myocardial infarction	5 (0.2)	54 (0.2)	0.9
Other ischemic heart disease	28 (1.0)	167 (0.6)	**0.01** ^b^
Hypertension	104 (3.6)	406 (1.4)	**<0.001**
Heart failure	9 (0.3)	67 (0.2)	0.4
Sudden cardiovascular death	0 (0.0)	0 (0.0)	1.0
Non-haemorrhagic stroke	8 (0.3)	90 (0.3)	0.8
Chronic kidney disease	15 (0.5)	44 (0.2)	**<0.001**
Type 2 diabetes	29 (1.0)	224 (0.8)	0.2
**Autoimmune conditions**			
Type 1 diabetes	48 (1.7)	211 (0.7)	**<0.001**
Coeliac disease	41 (1.4)	193 (0.7)	**<0.001**
Autoimmune thyroiditis	9 (0.3)	28 (0.1)	**0.001**
Autoimmune alopecia	12 (0.4)	40 (0.1)	**<0.001**
Multiple sclerosis	5 (0.2)	49 (0.2)	1.0

a Chi-square test.

b Non-significant after Bonferroni correction.

JIA: juvenile idiopathic arthritis. Significant values in bold. Significance level is *P* < 0.05.

### Autoimmune conditions

Adults with JIA had significantly higher 5-year retrospective prevalence (%) of the autoimmune diseases type 1 DM (1.7 *vs* 0.7), coeliac disease (1.4 *vs* 0.7), autoimmune thyroiditis (0.3 *vs* 0.1) and autoimmune alopecia (0.4 *vs* 0.1) (Table 2, Fig. 2). As expected, adults with JIA also had a significantly higher prevalence (%) of uveitis (6.4 *vs* 0.1), psoriasis (4.3 *vs* 0.7) and IBD (2.3 *vs* 0.7).

### Charlson Comorbidity Index (CCI)

Overall, JIA patients above 25 years of age had a significantly higher CCI score than comparators [mean (SD) 0.25 (0.83) *vs* 0.16 (0.65)]. When stratifying into 10-year age groups according to age when 5-year retrospective data first became available, the differences remained statistically significant in the age groups 35–44 years, 45–54 years and 55–64 years (Table 3).

**Table 3:** Charlson Comorbidity Index (CCI) score in adults with JIA and comparators 5 years retrospectively.

	Adults with JIA			Comparators			
Age (yrs)	Median (IQR)	Mean (SD)	N	Median (IQR)	Mean (SD)	N	***P* value** ^a^
**25–34**	0 (0**–**0)	0.07 (0.33)	467	0 (0**–**0)	0.05 (0.29)	4618	0.3
**35–44**	0 (0**–**0)	0.26 (0.92)	351	0 (0**–**0)	0.09 (0.47)	3552	**<0.001**
**45–54**	0 (0**–**0)	0.23 (0.64)	195	0 (0**–**0)	0.15 (0.61)	1964	**<0.05**
**55–64**	0 (0**–**1)	0.61 (1.27)	93	0 (0**–**0)	0.30 (0.86)	938	**<0.05**
**65–74**	0 (0**–**2)	1.16 (1.93)	45	0 (0**–**1)	0.71 (1.36)	511	0.1
**75–84**	0 (0**–**1)	0.52 (0.75)	27	0 (0**–**2)	1.02 (1.60)	300	0.3
**≥85**	0 (0**–**1)	0.44 (0.73)	9	0 (0**–**1)	0.93 (1.27)	126	0.4
**Total**	0 (0**–**0)	0.25 (0.83)	1187	0 (0**–**0)	0.16 (0.65)	12 009	**<0.001**

a Group differences analysed using the Mann–Whitney *U* test. Significant *P*-values are highlighted in **bold**.

JIA: juvenile idiopathic arthritis; yrs: years; SD: standard deviation; IQR: interquartile range (25th–75th quartile).

### All-cause mortality

During a follow-up time of ∼15 years, all-cause mortality was significantly higher in JIA compared with comparators [mortality rate JIA *vs* comparators: 2.39 *vs* 1.80 per 1000 person-years at risk, JIA hazard ratio (HR) (95% CI) 1.33 (1.03–1.72)] (Fig. 3). Adjusting for centrality index and educational level did not affect significance levels nor the magnitude of the findings (data not shown).

**Figure 3:**
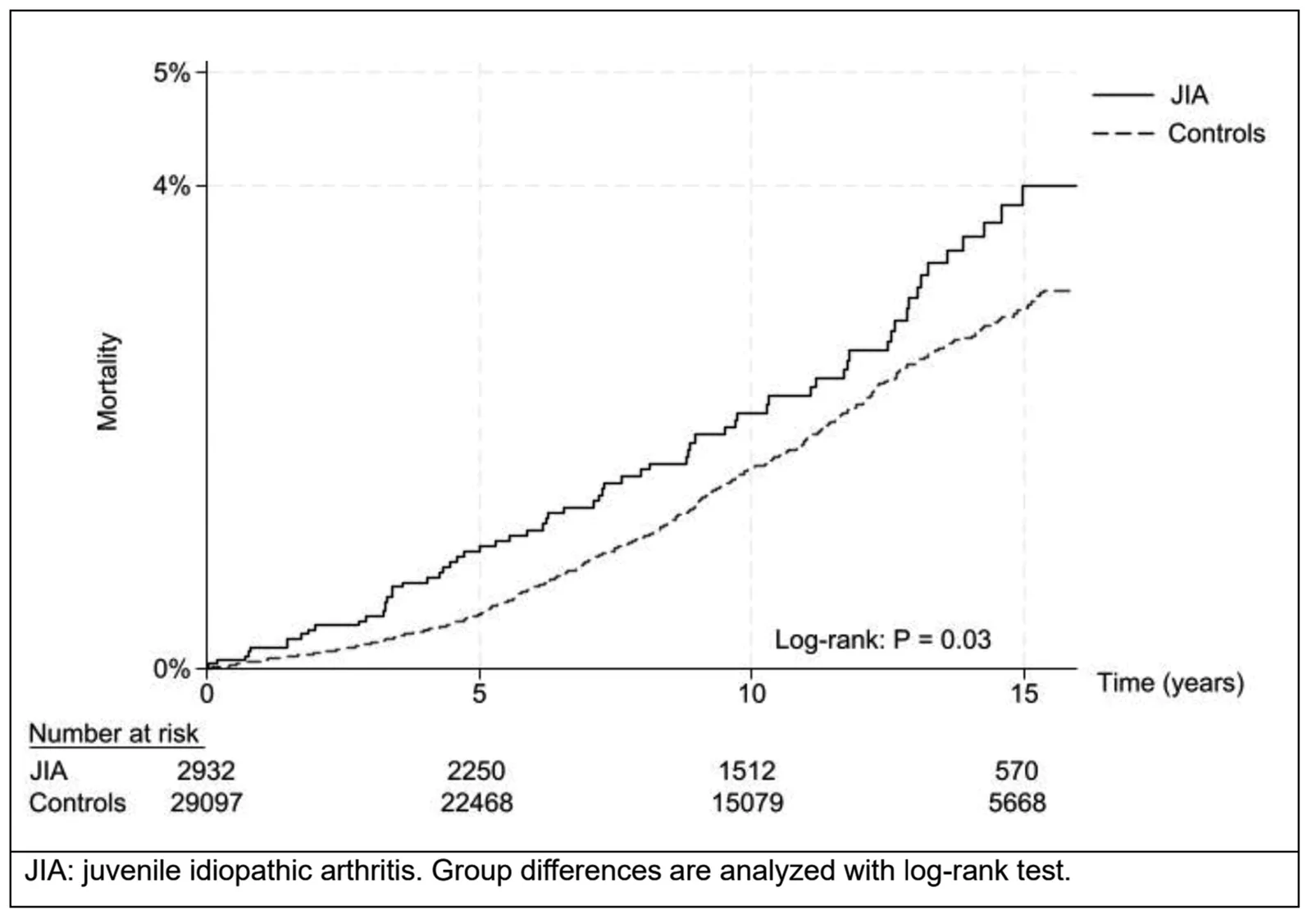
Kaplan–Meier analysis of cumulative mortality in adults with JIA and comparators

### Outcomes in adult JIA with *vs* without recent or current DMARD treatment

In adult JIA with *vs* without recent or current DMARD exposure there were no differences in comorbidity prevalences after adjusting for age and gender. Patients recently or currently using DMARDs had a higher prevalence of psoriasis and uveitis (Supplementary Table S3). The overall modified CCI score was significantly higher in JIA patients who were not recently or currently treated with DMARDs; however, there were no differences when stratifying the population by gender and 10-year age groups (Supplementary Table S4 and S5). The unadjusted risk of all-cause mortality was significantly higher in JIA not recently or currently exposed to DMARDs at the index date (*P* = 0.03, log-rank test) (Supplementary Fig. S1); however, the risk was no longer statistically significant after adjusting for age and gender [HR (95% CI): 0.77 (0.43–1.36)] (Supplementary Table S6).

### Differences in comorbidity prevalences in those included before *vs* in 2014 or later

When analysing outcomes between JIA and comparators stratified by inclusion before *vs* in 2014 or later, differences in prevalence of other ischemic heart disease, hypertension and autoimmune thyroiditis were only significant among those included before 2014. In contrast, differences in coeliac disease were only significant in those included after 2014, while differences in chronic kidney disease, type 1 diabetes and autoimmune alopecia remained significant regardless of inclusion period (Supplementary Table S7). In analyses of adults with JIA with *vs* without recent or current DMARD exposure, results were similar regardless of time of inclusion (Supplementary Table S8).

## Discussion

This study indicates a higher prevalence of comorbidities and autoimmune diseases and a higher mortality rate in adults with JIA compared with age- and gender-matched comparators. Outcomes were similar in adults with JIA with and without recent or current DMARD treatment. To our knowledge, no previous study has explored the prevalence of comorbidities and autoimmune diseases and all-cause mortality in adult JIA compared with matched comparators, and in adult JIA with *vs* without recent or current DMARD exposure.

There was a significantly higher prevalence of ischemic heart disease (excluding acute MI), hypertension and CKD in adults with JIA, possibly explaining the higher CCI score in age groups 35–44, 45–54 and 55–64 years. Low prevalences in the youngest subpopulation and small sample sizes of older patients may have provided insufficient power to detect statistical differences. A previous study found no elevated cardiovascular risk in adults with non-systemic JIA [8]. However, the study explored self-reported comorbidities and had a substantially smaller JIA population than our study. Our results are not unexpected given the higher risk of CVD [2, 3] and CKD [4] in adult-onset IJDs and the longer disease duration in adults with JIA.

Adults with JIA had a higher prevalence of type 1 DM, coeliac disease, autoimmune thyroiditis and autoimmune alopecia, in line with a previous study exploring the autoimmune burden in 79 JIA patients aged 3–21 years [21]. Coeliac disease and type 1 DM have also been linked to JIA elsewhere [34]. The finding of an increased prevalence of autoimmune alopecia in adult JIA is novel as this association has not been explored previously, likely due to underreporting of alopecia. Multiple sclerosis has been linked to JIA in a published literature review [35]; however, this association was not found in our study and the current evidence is limited. The higher prevalences of psoriasis, uveitis and IBD in JIA are expected as these are extraarticular manifestations of JIA [16].

On the basis that adults with JIA had a higher prevalence of CVD, CKD, type 1 DM and coeliac disease, as well as a higher overall modified CCI score, the finding of a higher all-cause mortality in adults with JIA was not wholly unexpected [32]. Individually, CVD, CKD, type 1 DM and coeliac disease are associated with increased all-cause mortality in the general population [36–39]. Studies exploring mortality in children with JIA have conflicting results [40, 41]. Since adults with JIA remain a relatively young population in published registry studies [7, 8, 17, 19, 20, 42–44], there is a lack of previous large studies exploring long-term outcomes in adult JIA, and mortality risk estimation is subject to uncertainty. Our study is the first with a large population to explore all-cause mortality in adults with JIA.

Our findings also indicate that adults with JIA with and without recent DMARD exposure have a similar prevalence of comorbidities, as well as a similar risk of all-cause mortality. Psoriasis and uveitis give an added indication for DMARD treatment in adults with JIA, explaining the higher prevalence of these conditions in those recently or currently exposed to DMARDs. However, two previous findings are not confirmed in our study: (i) the association between methotrexate and CKD and hypertension in a previous study of children with JIA [45] and (ii) the higher exposure to NSAIDs in adults with JIA *vs* comparators, given the well-known risk profile of NSAIDs and their association with CKD. Notably, over-the-counter purchases of NSAIDs are not registered in the Norwegian prescribed drug registry and detected group differences of NSAID exposure are likely overestimated.

Our study has limitations. Using ICD-10 code-based case identification, adults diagnosed with JIA in childhood and not receiving specialist healthcare services at any time during the study period—for example, due to persistent drug-free remission—are not included in the study. This causes a selection bias towards patients with active disease that requires follow-up and results must be interpreted accordingly. However, the primary aim of our study was to explore outcomes in adults with active JIA in adulthood. Long-term follow-up data of patients with JIA from childhood and into adulthood would be valuable and would also increase the validity of the adult JIA population; however, we did not have access to diagnosis codes in individuals below 18 years of age.

By redefining index dates to 1 January 2014 for meeting the inclusion criteria between 2009 and 2014, there is a risk for biasing findings, which may be supported by the alteration of results when stratifying analyses on comorbidity prevalences by time of inclusion. However, the alteration of results can have many possible explanations, and we deemed it necessary to include the whole population in the analyses to obtain sufficient statistical power. Still, the potential for bias must be considered when interpreting the results.

Detection bias of CVD in adults with JIA is possible, as the awareness on risk identification of CVD in IJDs has increased among rheumatologists during the period of this study [46]. The European Alliance of Associations for Rheumatology (EULAR) recommends that the rheumatologist is responsible for CVD risk management in patients with IJDs [47]. In Norway, there has been a national initiative among all rheumatology departments for CVD risk identification in individuals with IJDs [48]. Thus, adults with IJDs are more likely to receive diagnoses of established CVD risk factors within specialist care. In contrast, comparators are more likely to remain undiagnosed or receive such diagnoses in primary care, where they are not captured in the NPR, thus not included in our data.

Also, the lack of data on disease activity and reasons for not using DMARDs need to be considered when interpreting the results. The definition of DMARD exposure, being registered dispensations of DMARD prescription or DMARD infusions during the year up to and including the index date, is not optimal; however, this was considered an appropriate approach given the limitations of the data available.

Furthermore, diagnosis coding is physician-dependent and can be influenced by individual and local practices. However, the inclusion criterium of at least two ICD-10 codes of non-systemic JIA gives a positive predictive value of 93.4% according to a recent study assessing JIA coding in children [49]. The same study estimated an incidence of JIA in Norway corresponding to 144 new cases per year, nearly identical to the median number of adult JIA patients fulfilling the inclusion criteria each year in our study. Hence, we are confident that the inclusion criterium for JIA in adulthood in this study is reliable. Combined with a high-quality Norwegian healthcare system with complete nationwide registers [50], this strengthens the validity of our study population.

Lack of precision in coding may have contributed to the low number of older adults with JIA. Historically, adults with JIA have probably been more likely to receive a wrongful diagnosis of an adult-onset IJD during follow-up, as the awareness of JIA in adulthood only recently has increased. However, this underrepresentation of older age groups likely did not affect the comparative analyses due to age matching. Still, as the included non-autoimmune comorbidities are age-dependent, our study likely underestimates the lifetime prevalence of these diseases in JIA and comparators. To support our findings, large population-based studies with an age structure more comparable to that of the general population are needed. Longitudinal studies including patients at the peak age for comorbidity incidence would give valuable information on the lifetime comorbidity risk in adults with JIA.

To conclude, in this nationwide register study from Norway, adults with JIA have a higher prevalence of common comorbidities and autoimmune diseases, as well as an increased all-cause mortality compared with age-and gender-matched comparators from the general population. Comorbidity prevalences and all-cause mortality was similar in adults with JIA with and without recent or current DMARD exposure. Our study adds important information about the disease burden of JIA in adulthood. Comorbidity screening should be considered as part of the routine follow-up of this patient group.

## Supplementary Material

keag507_Supplementary_Data

## Data Availability

The data underlying this article are available in the article and in its online supplementary material. NPR data can be accessed here: https://helsedata.no/en/forvaltere/norwegian-institute-of-public-health/norwegian-patient-registry-npr/
