# Supplementary material for: Comorbidity and mortality in adults with juvenile idiopathic arthritis: a nationwide Norwegian register-based matched cohort study
Source: Rheumatology (Oxford). 2026 Sep 15;65(10):keag507. doi: 10.1093/rheumatology/keag507 (PMC13633849; doi:10.1093/rheumatology/keag507)
Supplement: Supplementary file 1 [file keag507_supplementary_data.docx]

**SUPPLEMENTARY MATERIAL**

| **Supplementary table S1.** ICD-10 codes used to define comorbidities and autoimmune conditions | |
| --- | --- |
| **Disease/condition** | **ICD-10 codes** |
| Non-autoimmune |  |
| Acute myocardial infarction | I21-I22 |
| Other ischemic heart disease | I20, I23-I25 |
| Hypertension | I10-I13 |
| Heart failure | I11.0, I13.0, I50 |
| Sudden cardiac death | I46.1 |
| Non-hemorrhagic stroke | I60, I61, I63, I64, H31.4, G45 |
| Chronic kidney disease | N18 |
| Type 2 diabetes mellitus | E11 |
| Autoimmune |  |
| Type 1 diabetes mellitus | E10 |
| Celiac disease | K90.0 |
| Autoimmune thyroiditis | E06.3 |
| Autoimmune alopecia | L63 |
| Multiple sclerosis | G35 |
| Psoriasis | L40 |
| Crohn’s disease | K50 |
| Ulcerative colitis | K51 |
| Uveitis | H20.0 |
| ICD-10: International Statistical Classification of Diseases and Related Health Problems, 10^th^ Revision | |

| **Supplementary table S2.** Overview of individual drugs within each DMARD subgroup and ATC code | |
| --- | --- |
| **DMARD subgroup** | **ATC code** |
| **csDMARDs** | |
| Methotrexate | L04A X03 |
| Leflunomide | L04A K01 |
| Sulfasalazine | A07E C01 |
| **bDMARDs** | |
| Abatacept | L04A A24 |
| Adalimumab | L04A B04 |
| Anakinra | L04A C03 |
| Bimekizumab^a^ | L04A C21 |
| Canakinumab^a^ | L04A C08 |
| Certolizumab | L04A B05 |
| Etanercept | L04A B01 |
| Golimumab | L04A B06 |
| Guselkumab^a^ | L04A C16 |
| Iksekizumab^a^ | L04A C13 |
| Infliximab | L04A B02 |
| Rituximab | L0F1 A01 |
| Risankizumab^a^ | L04A C18 |
| Sekukinumab | L04A C10 |
| Tocilizumab | L04A C07 |
| Ustekinumab | L04A C05 |
| **tsDMARDs** | |
| Baricitinib | L04A F02 |
| Filgotinib^a^ | L04A F04 |
| Tofacitinib^a^ | L04A F01 |
| Upadacitinib^a^ | L04A F03 |
| DMARD: disease-modyfing anti-rheumatic drug; csDMARD: conventional synthetic DMARD; bDMARD: biologic DMARD; tsDMARD: targeted synthetic DMARD; ATC: anatomical therapeutic chemical classification system. ^a^No patient was registered as treated with these drugs during the year prior to index date. | |

| **Supplementary table S3***.* 5-year retrospective prevalence (n(%)) of comorbidities in JIA with and without exposure to DMARDs | | | |
| --- | --- | --- | --- |
|  | **DMARDs**  **N = 1525** | **No DMARDs**  **N = 1371** | **Age- and gender adjusted OR (95% CI)** |
| **Comorbidities** |  |  |  |
| Myocardial infarction | 1 (0.1) | 4 (0.3) | 0.4 (0.0 – 4.4) |
| Other ischemic heart disease | 9 (0.6) | 19 (1.4) | 1.2 (0.5 – 3.0) |
| Hypertension | 37 (2.4) | 67 (4.9) | 1.3 (0.8 – 2.1) |
| Heart failure | 0 (0.0) | 9 (0.7) | 1.0 |
| Sudden cardiovascular death | 0 (0.0) | 0 (0.0) | 1.0 |
| Non-hemorrhagic stroke | 2 (0.1) | 6 (0.4) | 0.9 (0.2 – 5.3) |
| Chronic kidney disease | 3 (0.2) | 12 (0.9) | 0.4 (0.1 – 1.5) |
| Type 2 diabetes | 8 (0.5) | 21 (1.5) | 0.6 (0.3 – 1.5) |
| **Autoimmune conditions** |  |  |  |
| Type 1 diabetes | 23 (1.5) | 25 (1.8) | 0.9 (0.5 – 1.6) |
| Celiac disease | 24 (1.6) | 17 (1.2) | 1.1 (0.6 – 2.1) |
| Autoimmune thyroiditis | 5 (0.3) | 4 (0.3) | 1.1 (0.3 – 4.2) |
| Autoimmune alopecia | 5 (0.3) | 7 (0.5) | 0.6 (0.2 – 1.9) |
| Multiple sclerosis | 2 (0.1) | 3 (0.2) | 0.8 (0.1 – 5.1) |
| Psoriasis | 85 (5.6) | 38 (2.8) | **2.2 (1.5 – 3.3)** |
| Inflammatory bowel disease | 43 (2.8) | 24 (1.8) | 1.5 (0.9 – 2.5) |
| Crohn’s disease | 33 (2.2) | 15 (1.1) | 1.8 (0.9 – 3.3) |
| Ulcerative colitis | 19 (1.3) | 14 (1.0) | 1.1 (0.6 – 2.3) |
| Uveitis | 130 (8.5) | 54 (3.9) | **1.9 (1.4 – 2.7)** |
| JIA: juvenile idiopathic arthritis; DMARDs: disease-modifying anti-rheumatic drugs; OR: Odds ratio; CI: confidence interval. | | | |

| **Supplementary table S4.** CCI-score in adult females with JIA exposed vs. not exposed to DMARDs, based on 5-year retrospective data | | | | | | | |
| --- | --- | --- | --- | --- | --- | --- | --- |
|  | **JIA exposed to DMARDs** | | | **JIA not exposed to DMARDs** | | |  |
| **Age (yrs)** | **Median (IQR)** | **Mean (SD)** | **N** | **Median (IQR)** | **Mean (SD)** | **N** | **P***** |
| **25-34** | 0 (0-0) | 0.03 (0.16) | 152 | 0 (0-0) | 0.1 (0.39) | 201 | 0.1 |
| **35-44** | 0 (0-0) | 0.26 (0.81) | 115 | 0 (0-0) | 0.31 (1.13) | 166 | 0.3 |
| **45-54** | 0 (0-0) | 0.13 (0.47) | 56 | 0 (0-0) | 0.27 (0.72) | 96 | 0.2 |
| **55-64** | 0 (0-0) | 0.31 (0.62) | 26 | 0 (0-1) | 0.67 (1.35) | 51 | 0.4 |
| **65-74** | 1 (0-2) | 1.00 (1.12) | 9 | 0 (0-1) | 1.15 (2.03) | 27 | 0.6 |
| **75-84** | - | - | 1 | 0 (0-1) | 0.52 (0.81) | 21 | - |
| **≥ 85** | - | - | 0 | 0 (0-1) | 0.29 (0.49) | 7 | - |
| **Total** | 0 (0-0) | 0.16 (0.58) | 359 | 0 (0-0) | 0.31 (0.97) | 569 | **< 0.05** |
| CCI: Charlson Comorbidity Index; JIA: juvenile idiopathic arthritis; DMARDs; disease-modifying anti-rheumatic drugs; IQR: interquartile-range; SD: Standard deviation. *Mann-Whitney U-test. | | | | | | | |

| **Supplementary table S5.** CCI-score in adult males with JIA exposed vs. not exposed to DMARDs, based on 5-year retrospective data | | | | | | | |
| --- | --- | --- | --- | --- | --- | --- | --- |
|  | **JIA exposed to DMARDs** | | | **JIA not exposed to DMARDs** | | |  |
| **Age (yrs)** | **Median (IQR)** | **Mean (SD)** | **N** | **Median (IQR)** | **Mean (SD)** | **N** | **P value^a^** |
| **25-34** | 0 (0-0) | 0.09 (0.36) | 45 | 0 (0-0) | 0.06 (0.38) | 69 | 0.6 |
| **35-44** | 0 (0-0) | 0.06 (0.25) | 32 | 0 (0-0) | 0.16 (0.49) | 38 | 0.5 |
| **45-54** | 0 (0-0) | 0.24 (0.56) | 17 | 0 (0-0) | 0.31 (0.74) | 26 | 0.9 |
| **55-64** | 0 (0-1) | 0.33 (0.52) | 33 | 0 (0-3) | 1.3 (2.1) | 10 | 0.6 |
| **65-74** | - | - | 1 | 0 (0-3) | 1.5 (2.51) | 8 | - |
| **75-84** | 0 (0-1) | 0.33 (0.58) | 3 | 1 (1-1) | 1 (0) | 2 | 0.6 |
| **≥ 85** | - | - | 0 | 1 (0-2) | 1 (1.41) | 2 | - |
| **Total** | 0 (0-0) | 0.13 (0.39) | 104 | 0 (0-0) | 0.30 (0.97) | 155 | 0.4 |
| CCI: Charlson Comorbidity Index; JIA: juvenile idiopathic arthritis; DMARDs; disease-modifying anti-rheumatic drugs; IQR: interquartile-range; SD: Standard deviation. ^a^Mann-Whitney U-test. | | | | | | | |

**Supplementary figure S1**. All-cause mortality in JIA treated vs. not treated with DMARDs the last year

JIA: juvenile idiopathic arthritis; DMARDs: disease-modifying anti-rheumatic drugs

| **Supplementary table S6.** Cox proportional hazards regression models of all-cause mortality in adults with JIA | | |
| --- | --- | --- |
|  | **Variables** | **HR (95% CI)** |
| **Model 1** | Age, gender, DMARD exposure | **0.77 (0.43 – 1.36)** |
| **Model 2** | Age, gender centrality index, educational level, DMARD exposure | **0.93 (0.47 – 1.85)** |
| **Model 3** | Age, gender, 5-year retrospective CCI score, DMARD exposure | **0.83 (0.46 – 1.48)** |
| JIA: juvenile idiopathic arthritis; HR: hazard ratio; DMARD: disease-modifying anti-rheumatic drugs; CCI: Charlson comorbidity index | | |

| **Supplementary table S7.** P-values for difference in 5-year retrospective comorbidity prevalences between adults with JIA vs. population comparators included before and after 2014 | | |
| --- | --- | --- |
|  | **Included before 2014**  **N = 15 180** | **Included in 2014 or later**  **N = 17 341** |
| **Comorbidities** |  |  |
| Myocardial infarction | 0.98 | 0.79 |
| Other ischemic heart disease | **0.02** | 0.22 |
| Hypertension | **<0.001** | 0.11 |
| Heart failure | 0.77 | 0.06 |
| Sudden cardiovascular death | - |  |
| Non-hemorrhagic stroke | 0.59 | 0.78 |
| Chronic kidney disease | **<0.001** | **0.01** |
| Type 2 diabetes | 0.65 | 0.11 |
| **Autoimmune conditions** | | |
| Type 1 diabetes | **0.002** | **<0.001** |
| Celiac disease | 0.07 | **<0.001** |
| Autoimmune thyroiditis | **<0.001** | 0.37 |
| Autoimmune alopecia | **0.01** | **0.02** |
| Multiple sclerosis | 0.81 | 0.65 |
| JIA: juvenile idiopathic arthritis | | |

| **Supplementary table S8.** Odds Ratios (OR) for differences in comorbidities and autoimmune conditions between adults with JIA with vs. without recent/current DMARD exposure, in total and divided into patients included before vs. in 2014 or later | | | |
| --- | --- | --- | --- |
|  | **Age- and gender adjusted OR (95% CI)** | **Age-and gender adjusted OR before 2014 (95% CI)** | **Age-and gender adjusted OR included in 2014 or later (95% CI)** |
| **Comorbidities** |  |  |  |
| Myocardial infarction | 0.4 (0.0 – 4.4) | 0.5 (0.0 – 4.8) | -* |
| Other ischemic heart disease | 1.2 (0.5 – 3.0) | 1.9 (0.7 – 5.3) | -* |
| Hypertension | 1.3 (0.8 – 2.1) | 1.0 (0.6 – 1.6) | 2.0 (0.5 – 8.0) |
| Heart failure | 1.0 | 1.0 | 1.0 |
| Sudden cardiovascular death | 1.0 | 1.0 | 1.0 |
| Non-hemorrhagic stroke | 0.9 (0.2 – 5.3) | 1.3 (0.2 – 9.2) | -* |
| Chronic kidney disease | 0.4 (0.1 – 1.5) | 0.3 (0.1 – 1.6) | 0.6 (0.1 – 6.8) |
| Type 2 diabetes | 0.6 (0.3 – 1.5) | 1.0 (0.4 – 2.9) | 0.2 (0.0 – 1.4) |
| **Autoimmune conditions** |  |  |  |
| Type 1 diabetes | 0.9 (0.5 – 1.6) | 0.7 (0.3 – 1.6) | 1.3 (0.5 – 3.1) |
| Celiac disease | 1.1 (0.6 – 2.1) | 0.6 (0.2 – 1.9) | 1.1 (0.5 – 2.4) |
| Autoimmune thyroiditis | 1.1 (0.3 – 4.2) | 1.0 (0.2 – 5.1) | 1.0 (0.1 – 11.4) |
| Autoimmune alopecia | 0.6 (0.2 – 1.9) | 1.0 (0.2 – 5.0) | 0.4 (0.1 – 2.1) |
| Multiple sclerosis | 0.8 (0.1 – 5.1) | 0.5 (0.0 – 5.5) | 2.6 (0.0 – 85.0) |
| Psoriasis | **2.2 (1.5 – 3.3)** | **2.4 (1.4 – 4.2)** | **2.0 (1.1 – 3.6)** |
| Inflammatory bowel disease | 1.5 (0.9 – 2.5) | 1.1 (0.5 – 2.2) | 2.1 (0.9 – 4.9) |
| Crohn’s disease | 1.8 (0.9 – 3.3) | 1.1 (0.5 – 2.6) | 3.1 (1.1 – 8.7) |
| Ulcerative colitis | 1.1 (0.6 – 2.3) | 1.2 (0.5 – 2.8) | 0.8 (0.2 – 3.1) |
| Uveitis | **1.9 (1.4 – 2.7)** | **2.0 (1.2 – 3.2)** | **1.9 (1.2 – 3.0)** |
| JIA: juvenile idiopathic arthritis; DMARDs: disease-modifying anti-rheumatic drugs; OR: Odds ratio; CI: confidence interval. * Too low sample size to generate OR. | | | |
